# Role of Antioxidants in Alleviating Bisphenol A Toxicity

**DOI:** 10.3390/biom10081105

**Published:** 2020-07-25

**Authors:** Shehreen Amjad, Md Saidur Rahman, Myung-Geol Pang

**Affiliations:** Department of Animal Science & Technology and BET Research Institute, Chung-Ang University, Anseong, Gyeonggi-do 17546, Korea; shehreenamjad@gmail.com (S.A.); shohagvet@gmail.com (M.S.R.)

**Keywords:** bisphenol A, antioxidants, diseases, stress, endocrine disruptors

## Abstract

Bisphenol A (BPA) is an oestrogenic endocrine disruptor widely used in the production of certain plastics, e.g., polycarbonate, hard and clear plastics, and epoxy resins that act as protective coating for food and beverage cans. Human exposure to this chemical is thought to be ubiquitous. BPA alters endocrine function, thereby causing many diseases in human and animals. In the last few decades, studies exploring the mechanism of BPA activity revealed a direct link between BPA-induced oxidative stress and disease pathogenesis. Antioxidants, reducing agents that prevent cellular oxidation reactions, can protect BPA toxicity. Although the important role of antioxidants in minimizing BPA stress has been demonstrated in many studies, a clear consensus on the associated mechanisms is needed, as well as the directives on their efficacy and safety. Herein, considering the distinct biochemical properties of BPA and antioxidants, we provide a framework for understanding how antioxidants alleviate BPA-associated stress. We summarize the current knowledge on the biological function of enzymatic and non-enzymatic antioxidants, and discuss their practical potential as BPA-detoxifying agents.

## 1. Introduction

Bisphenol A [BPA; (CH_3_)_2_C(C_6_H_4_OH)_2_] is an organic synthetic compound and a major environmental pollutant. It is widely used to manufacture numerous consumer products, including food packaging materials, industrial supplies, dental sealant, and others. BPA is an endocrine disruptor. It interferes with hormone function via oestrogenic, anti-androgenic, and anti-thyroid activity [1,2,3,4]. Exposure to this chemical is ubiquitous, and occurs mostly via the oral (approximately 90%), respiratory, and dermal routes in human and animals [5,6]. In addition, BPA is reportedly passed from the mother to offspring by intrauterine transmission during prenatal embryonic development and via breastfeeding during the early neonatal period [7]. 

Based on the available evidence, BPA has a very weak binding affinity for certain hormonal receptors. As an example, the binding affinity of BPA to oestrogen receptor is 1000–10,000 times weaker than that of natural oestrogen (diethylstilbestrol) [8]. Normal endocrine signalling does not affect the overall hormone levels to a great extent. However, even small alterations of hormone function can potentially greatly affect biological activity [9]. Consequently, scientists believe that BPA may impact the delicate endocrine balance, leading to diverse pathological outcomes. Interestingly, BPA was initially used as a growth promoter in the cattle and poultry industries, but was later proved to be highly toxic [10]. Specifically, exposure to BPA is linked to cardiovascular disease, brain development abnormalities, obesity, hypertension, thyroid dysfunction, diabetes, breast cancer, infertility, etc., in human, and terrestrial and aquatic animals, as summarized by Rochester [11].

Harmful effects of BPA in cells and tissues (both in vitro and in vivo) are mostly mediated by increased oxidative stress associated with an elevated production of toxic free radicals, in addition to the classical genomic and non-genomic mechanisms of activity [4,5]. Although intracellular free radicals, most importantly, reactive oxygen species (ROS), are critical regulators of cellular physiology, their increased levels can directly affect DNA, RNA, and proteins, subsequently predisposing the cell to pathology [12,13]. 

By contrast, an antioxidant is a reducing agent that scavenges and neutralizes free radicals, thereby preventing oxidation reactions. Indeed, the important role of enzymatic antioxidants, e.g., superoxide dismutase (SOD), catalase (CAT), or glutathione (GSH) system, in overcoming the harmful effects of BPA has been highlighted in many studies. Simultaneously, the significance of non-enzymatic antioxidants in the biochemistry of living organism has been uncovered in the recent decades. For instance, Lobo et al. [14] reported that vitamin E prevents peroxidation of lipids by scavenging ROS before they damage the cell. Similar, the function of germ cells, neuronal, and kidney cells is preserved by antioxidants during BPA co-exposure and/or administration both in vitro and in vivo [13,15,16,17,18]. Importantly, antioxidants safely interact with free radicals generated upon BPA exposure and terminate the oxidation chain reaction before vital molecules, such as DNA, RNA, and proteins, are damaged [14]. It is tempting to speculate that antioxidants could be used as a potential defence and/or treatment regime against BPA toxicity. 

Considering the specific biochemical properties of BPA and antioxidants, herein, we focus on how antioxidants can be used to overcome BPA toxicity, as reported in in vitro scenarios, and in studies involving animals and human subjects. We first provide a brief overview of the modes of action of BPA and antioxidants, followed by detailed descriptions of enzymatic and non-enzymatic antioxidants, and their utility as BPA-detoxifying agents.

## 2. Overview of BPA Activity

As an endocrine disrupting chemical, BPA acts as selective modulator of oestrogen receptor (ER), activating oestrogen-related receptor gamma and growth factor receptors [1,3]. Further, it is an antagonist of the thyroid hormone receptor and possesses anti-androgenic properties [1]. 

**Figure 1:**
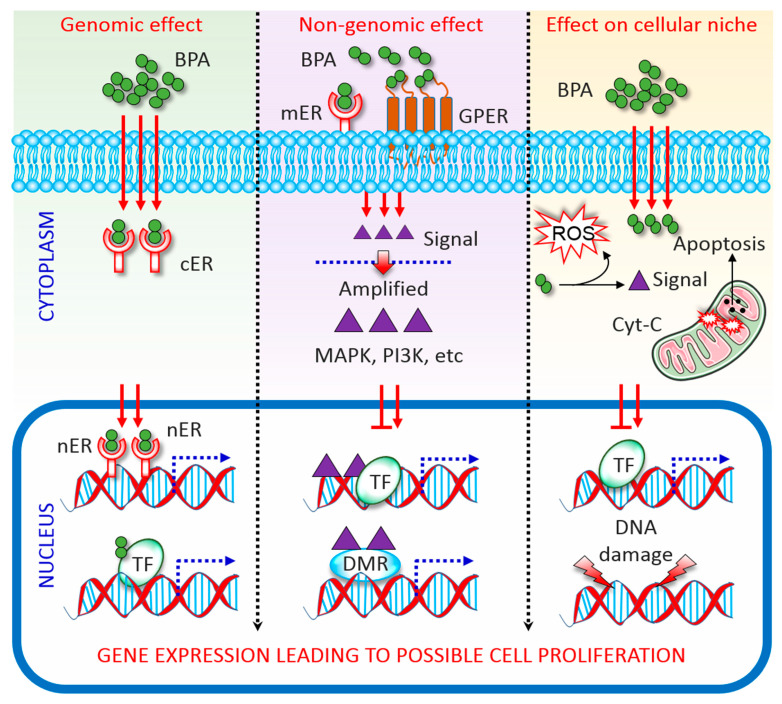
Mechanisms of Bisphenol A (BPA) activity. Genomic, non-genomic, and direct effects exerted by BPA are depicted. See the main text for details. BPA, bisphenol A; DMR, differentially methylated regions; cER, cytoplasmic oestrogen receptor; Cyt-C, cytochrome c; GPCR, G protein-coupled receptor; MAPK, mitogen-activated protein kinase; mER, membrane-bound oestrogen receptor; nER, nuclear oestrogen receptor; PI3K, phosphatidylinositol 3-kinase; ROS, reactive oxygen species; TF, transcription factor.

BPA has a structural similarity with oestrogen; therefore, capable of binding with both ERα and ERβ [4,18,19]. As an ER modulator, BPA acting via genomic and non-genomic pathways (Figure 1). In the genomic pathway, it binds to ER located in the cytoplasm (cER) or the nucleus (nER). The ER-BPA dimer binds to chromatin and activates transcription factors (TF), thereby leading to the transcription of target genes and affecting cell function [3,20]. In the non-genomic pathway, BPA binds to G protein-coupled receptors (GPCR) and cell membrane-bound ER (mER). Activation of both receptors triggers rapid oestrogen signalling via TF phosphorylation and activation of several kinase systems. In human ovarian cancer cells, such TF phosphorylation is mediated via the activation of mitogen-activated protein kinase (MAPK) and phosphatidylinositol 3-kinase, and changes in cAMP, protein kinase C, and protein kinase A levels that follow BPA binding to GPCR and mER [21]. It is important to note that the levels of circulating oestrogen greatly vary in males and females, which leading to the differential expression of ERs in different cells/tissues between both sexes. This is probably the major reason why BPA affect male and female differently [22]. On the other hand, BPA directly manipulates the cellular microenvironment. Plasma proteins that bind to BPA and protect the target cells from BPA can be changed upon BPA exposure. Detoxification of xenobiotics, including BPA, results in the formation of electrophiles, free radicals, nucleophiles, and redox-active reactants can destroy the DNA, RNA, and proteins [23]. Further, although the majority of BPA is metabolized to generate relatively less toxic BPA glucuronide and BPA sulphate, the remaining free BPA facilitates ROS formation via enzymatic and non-enzymatic reactions [24]. ROS reacting with nicotinamide adenine dinucleotide phosphate species concomitant with enzymatic processing facilitates the formation of diverse oxidative species, such as superoxides, peroxides, and hydroxyl radicals [24]. These free radicals lead to irreversible alteration of gene expression, apoptosis, and cell death. As such BPA interrupts cellular oxidative homeostasis by altering a dynamic balance between oxidative mediators and the activities of antioxidant enzymes, subsequently predispose to apoptosis [25,26]. 

Meli et al. [18] reported that BPA increases oxidative stress in the rat liver and spermatozoa by lowering the levels of antioxidant enzymes and increasing H_2_O_2_ and lipid peroxidation. These harmful effects were also capable of affecting the normal development of the kidney, brain, and testis as shown in mice model [18].

As an androgen receptor antagonist, BPA inhibits N- and C-terminal regions of the androgen receptor. This facilitates the interaction with a silencing mediator for thyroid hormone receptor and nuclear receptor co-repressor, subsequently suppressing the proliferation of Sertoli cells [27]. Although BPA-mediated effects on ER and androgenic receptor are relatively well studied, the mechanisms of BPA activity in different cell types/tissues, need to be investigated. 

## 3. Overview of Antioxidant Activity

Antioxidants are broadly classified into two major groups, such as enzymatic and non-enzymatic antioxidants. Both enzymatic and non-enzymatic antioxidants are capable of regulating the free radical reactions, subsequently, restore cellular integrity. Enzymatic antioxidants (e.g., SOD, CAT, and GSH) capable of converting oxidized metabolic products in a stepwise process to H_2_O_2_ and later to the water with the help of cofactors [28,29,30]. On the other hand, non-enzymatic antioxidants interrupt and terminate free radical chain reactions [31,32,33]. Examples of non-enzymatic antioxidants include vitamin E, vitamin A, vitamin C, flavonoids, carotenoids, glutathione, melatonin, and so on [33]. 

**Figure 2:**
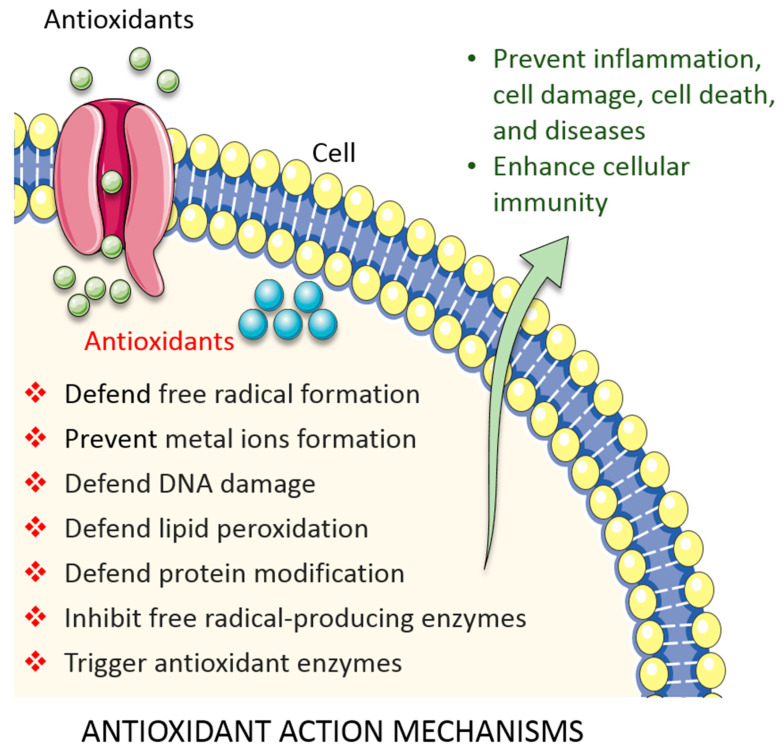
Overview of antioxidant defence systems in cell. See the main text for details.

In fact, different levels of antioxidant defences exist in living systems: radical-preventive, radical-scavenging, or radical-induced damage repair (Figure 2). First-line defence antioxidants rapidly neutralize any free radicals that would induce the production of other radicals or molecules that could become free radicals. The top three enzymes in this category are SOD, CAT, and GSH peroxidase. These enzymes decompose the superoxide radical, hydrogen peroxide (H_2_O_2_), and hydroperoxides, respectively, to harmless molecules (H_2_O or alcohol and O_2_). Metal ion-binding proteins, such as transferrin and caeruloplasmin, also represent this class of proteins; they bind to iron and copper, respectively, and prevent them from becoming free radicals [31,34]. Second-line defence antioxidants are typically scavenging antioxidants. They bind to active radicals, prevent chain reaction initiation, and break the chain propagation reactions. They donate an electron to free radicals to neutralize them and, in turn, become free radicals themselves albeit ones with reduced toxicity. These ‘new radicals’ are easily neutralized and rendered harmless by other antioxidants in this class. Examples include vitamin C, uric acid, and glutathione, which are hydrophilic, and alpha tocopherol (vitamin E) and ubiquinol, which are lipophilic [34]. Following free radical damage, third-line defence antioxidants become active. They are enzymes that repair the damage caused by free radicals, repairing damaged DNA, proteins, and lipids. Typical examples include DNA repair enzymes (polymerases, glycosylases, and nucleases) and proteolytic enzymes (proteinases, proteases, and peptidases), which are located both in the cytosol and mitochondrion in mammalian cell. Finally, fourth-line defence antioxidants prevent free radical formation and their reactions. The signal generated by free radicals induces the formation and transport of an appropriate antioxidant to the appropriate site [34].

## 4. Role of Antioxidants in Overcoming BPA Toxicity

A summary of the effects of antioxidants on BPA-induced stress in vivo and in vitro is presented in Table 1 and Table 2, respectively. In the current section, we provide a detailed description of how each antioxidant type helps the cell and/or tissue to overcome BPA toxicity.

### 4.1. Enzymatic Antioxidants

#### 4.1.1. SOD

SODs are enzymes that catalyse the conversion of superoxide radicals (O^2−^) to molecular oxygen (O_2_) and hydrogen peroxide (H_2_O_2_). They serve as a potent antioxidant defence against oxidative stress. SODs are found in almost all aerobic cells and extracellular fluids [35]. Three major residues (His48, His63, and Arg143) and two metal ions (Cu and Zn) are found in the active site of SOD, particularly SOD1. The nitrogen atoms of residue His63 bind to Cu and Zn ions. This binding is broken and re-formed during catalysis. Proper positioning and orientation of superoxide is required for electron transfer with Cu ion, and is achieved by Arg143. The active site of SOD is completely occupied by BPA via the formation of two hydrogen bonds traversing the substrate-binding cavity [36]. The interruption of the superoxide–copper interaction by BPA binding interferes with catalysis. Further, BPA forms many Van der Waal bonds with functionally important residues, including His48, His63, and Arg143, which prevents proper positioning of the superoxide in the active site. Consequently, BPA directly impairs the free radical-scavenging and enzymatic activities of SOD [36,37]. Ultimately, BPA exposure compromises cellular antioxidant defences by increasing ROS production [24].

Based on the available evidence, exposure to various doses of BPA severely damages the function of many vital organs and cells, e.g., the liver, kidney, pancreas, and testis, in animal models, which is accompanied by elevated ROS production associated with reduced SOD activity (reviewed in Gassman, [24]). BPA exposure also alters the expression of SOD2 in spermatogonial stem cells and spermatozoa in vitro [3,38], and in F1 male mouse during adulthood, following gestation exposure to BPA [4]. As a potential mitochondrial enzyme, SOD2 is extremely important in the regulation of mitochondrial death pathway and apoptosis signalling. Therefore, the altered expression of this enzyme in the BPA-exposed condition may be directly linked with abnormal growth and differentiation of target cells [39]. 

Alonso-Magdalena et al. [40,41] demonstrated that BPA-induce (single/short term exposure) stress to the male mice is associated with a declining blood glucose content and increased plasma insulin levels, subsequently predisposed to metabolic abnormalities, e.g., type 2 diabetes, hypertension, and dyslipidaemia. Indeed, increased oxidative stress is a key feature of metabolic diseases, therefore antioxidants play a potential role in these disease pathogenesis. As such altered antioxidant activities, including the activities of SOD, CAT, GSH, were reported in several investigations [39,40,41] that subsequently correlated with the development of various metabolic disorders, including diabetes and obesity. By contrast, no significant changes of SOD levels were reported in studies of ovarian cells from new-born mouse following either in utero or in vitro exposure to BPA [42,43]. That is probably because the BPA doses used (50 µg/kg bodyweight) were not sufficient to trigger and/or compromise antioxidant defences in mouse. Therefore, although SOD is very important for overcoming BPA-induced stress, further studies, particularly dose-dependent studies, are required to determine the appropriate BPA levels that trigger optimum SOD activity in different cells.

#### 4.1.2. CAT

CATs are a class of dismutase enzymes that facilitate decomposition of H_2_O_2_ to water and oxygen in the living cell. These enzymes are present in every organ of most known animals, with particularly high levels reported in the liver [14]. CATs prevent the oxidative radical-damaging effect of 17β oestradiol and diethylstilbestrol, and are highly efficient antioxidants [78]. Banerjee et al. [79] evaluated the effects of 9-d intraperitoneal administration of BPA to sexually mature female rats. BPA exposure significantly increased nitric oxide and lipid peroxidation levels in the ovarian granulosa cells, altered the levels of sex hormones associated with an increased pro-inflammatory cytokine activity (tumour necrosis factor α and interleukin 6), and significantly decreased CAT expression. In addition, pre-treatment of the female mice with a CAT-specific blocker 3-amino-1,2,4-triazole alongside BPA aggravated these effects. Therefore, CAT plays a major role in the functional integrity of ovarian granulosa cells and female reproductive performance upon BPA-mediated stress [79]. Moghaddam et al. [39] reported that exposure to BPA (0.5 and 2 mg/kg/day) for 4 weeks in male mice significantly increases circulating glucose levels and facilitate weight gain. BPA disrupts the dynamic balance of enzymatic antioxidants by decreasing the levels of SOD, GSH, and CAT in blood and pancreas [39,40,41]. Indeed, BPA-induced oxidative stress is responsible for adipose tissue increment subsequently leading to obesity-related metabolic syndrome. 

In another study, Kabuto et al. [47] reported that 5-d exposure of male mice to 25 and 50 mg BPA/kg/d significantly decreased CAT activity in the liver. However, the authors did not investigate whether the altered CAT activity was responsible for the functional alterations in the liver cells. Therefore, further studies are needed to evaluate CAT activity in different cells following BPA exposure in relation to its functional importance [47].

#### 4.1.3. GSH System

The GSH system is an enzymatic antioxidant system that plays a major role in the regulation of many important signalling pathways. It is a group of antioxidants (GSH, GSH reductase, GSH peroxidases, and GSH *S*-transferases) primarily responsible for removing ROS, reactive nitrogen species (RNS), and other electrophiles generated by xenobiotics [80]. GSH has thiol group that react with H_2_0_2_, hydroxyl radicals, and hydroperoxides forming alcohols. It is commonly found in animals, plants, and microorganisms [81]. GSH peroxidase is the most abundant and highly efficient scavenger of H_2_O_2_, and most active against lipid hydroperoxides [82].

Intraperitoneal injection of BPA (50 mg/kg/d) for 5 d into adult male mice significantly alters cellular GSH levels in several vital organs, including the brain, kidneys, and testis, because of an increased production of H_2_O_2_ [47]. Although the study authors did not establish whether BPA compromised antioxidant defences by altering GSH levels, clinical or subclinical status of these organs is conceivable. Recently, we have reported that gestational exposure of BPA (50 mg/kg bodyweight/d) significantly decreases the expression of GSH *S*-transferases in the spermatozoa of F1 male mice during adulthood [5]. The decreased GSH *S*-transferase levels were associated with fertility loss in male offspring, as evaluated by natural breeding with untreated female. In other studies, altered expression of both GSH *S*-transferases (decreased) and GSH peroxidase (increased) were reported in mature mouse spermatozoa following in vitro exposure to 1 µM BPA for 6 h. The altered antioxidant activities in spermatozoa were also associated with abnormal acrosome reaction and compromised the mitochondrial detoxification process, subsequently affecting the ability of spermatozoa to fertilize oocyte in an in vitro fertilization assay [3,6]. 

Moghaddam et al. [40,41] reported that BPA-exposed male mice have the tendency to increase fat deposition and weight gain by compromising antioxidant defence mechanisms (GSH activities alongside other enzymatic antioxidants) in the blood and pancreatic cells. Glutathione peroxidase and glutathione-S-transferase involved in potential defence against ROS and other oxygen-free radicals induced by BPA [40,41,83]. Although the role of the GSH system in overcoming BPA toxicity was suggested (as mentioned earlier), cell-specific effects of this potential antioxidant system upon BPA exposure should be investigated in future studies.

#### 4.1.4. Uric Acid

Adenine and guanine, nucleic acid constituents, are degraded to form uric acid, commonly found in the muscle, kidney, and liver [84]. Uric acid is an important low-molecular mass antioxidant present in biological fluids in human, birds, reptiles, and some primates [84,85]. As a potential antioxidant, it scavenges hydroxyl and peroxyl radicals in vitro and in vivo [84]. Antioxidant effects of uric acid have been demonstrated in diseases of the nervous system (such as Parkinson’s disease, multiple sclerosis, and acute stroke) and in cancer [86,87].

There is some evidence that BPA affects uric acid levels in vivo. For instance, oral administration of BPA for 6 weeks leads to increased serum uric acid levels in the rat kidney [50,83]. However, these changes do not affect kidney function. 

Xanthine oxidase plays an important role in the synthesis of hepatic uric acid. It catalyse last 2 oxidation reaction of purine degradation, which is flow limiting enzyme of uric acid synthesis in liver [49]. In mouse, BPA exposure enhances the body weight and activity of xanthine oxidase in the liver. Inhibition of xanthine oxidase with allopurinol results in decreased production of uric acid both in vivo and in vitro. Therefore, it is plausible that BPA enhances hepatic uric acid synthesis by activating xanthine oxidase [49]. However, many studies showed that BPA exposure also elicits increased uric acid levels in the heart [88,89]. 

### 4.2. Non-Enzymatic Antioxidants

#### 4.2.1. Vitamin C

Vitamin C (ascorbic acid) is an important non-enzymatic antioxidants. Human and other primate are incapable to synthesized vitamin C, therefore has to be gained from diet [90]. Fruits and vegetables are a prominent dietary source of vitamin C for human and animals. As a water-soluble antioxidant, it can potentially protect important biomolecules from damage by scavenging oxygen free radicals in cells, as has been shown in vitro and in vivo [91].

Vitamin C exerts a pro-oxidant effect, interacting with transition metal ions. Ferric iron (Fe^3+^) is converted into ferrous iron (Fe^2+^) in the presence of vitamin C, and Fe^2+^ reacts with H_2_O_2_ which forms hydroxyl radicals [92]. BPA affects iron metabolism in the kidney, but vitamin C treatment exerts a pro-oxidant effect interacting with iron [93]. It has been reported that 45-d BPA and vitamin C co-administration to male Wistar rats did not affect the body and organ weight compared to control group and it negatively affects hyperchromatic cell number in the brain cortex by increasing the oxidative stress elicited by BPA exposure. It is reported that vitamin C has pro-oxidant effects which can reduce metals to react with oxygen to form lipid peroxidation generator [94]. Bindhumol et al. [83] reported that 30-d oral administration of BPA significantly decreased the SOD, CAT, and GSH peroxidase activities in liver cells. These effects were alleviated by vitamin C co-administration [95]. In another study, vitamin C positively affected the concentration and motility of epididymal spermatozoa in rat exposed to BPA, by improving the function of enzymatic antioxidants and decreasing lipid peroxidation [51]. A similar protective effect was reported in mouse spermatozoa following in vitro exposure to vitamin C [13]. Treatment of BPA-exposed spermatozoa with vitamin C restored sperm motility by inhibiting the overproduction of cellular ROS, RNS, and ATP [13]. However, it did not enhance sperm fertilization ability. In the study, the doses of BPA and vitamin C were both 100 µM; hence, the vitamin C dose could be optimized to improve the sperm fertilizing ability. By contrast, Korkmaz et al. [96] showed that 60 mg vitamin C/kg/d does not prevent oxidative damage in the liver following BPA administration (25 mg/kg/d, three times/week for 50 d) in a rat model. Although an appropriate vitamin C level exerts a pronounced protective effect against oxidative stress, high vitamin C level has a pro-oxidant effect. Therefore, the above observations might be related to the pro-oxidant effect of vitamin C, as both aspartate transaminase and alanine transaminase levels were increased in the serum of treated rats [96]. Consistently, BPA exposure in rat increases malondialdehyde levels and decreases GSH levels in the brain [97]. Increased production of free oxygen radicals causes lipid peroxidation and oxidative stress in the brain cells of male rat [97]. Treatment with vitamin C does not prevent oxidative damage in the brain of male rat [98]. 

#### 4.2.2. Vitamin E

The major dietary sources of vitamin E are vegetables, cereals, meat, eggs, fruits, and wheat germ oil. It is also available as a dietary supplement. Vitamin E has potent cholesterol-lowering and antioxidant properties [99]. The therapeutic potential of vitamin E against prostate cancer has also been reported [100]. Because of its antithrombotic and anti-tumour effects, vitamin E can prevent cardiovascular disease, as reported by Theriault et al. [101]. 

Vitamin E is a group of compounds (tocopherols and tocotrienols). However, the effects of tocopherols have been studied most extensively because of the high bioavailability, ready absorption, and metabolism of these compounds [102]. Tocopherols react with lipid radicals produced in a lipid peroxidation chain reaction, protecting the cell membrane from oxidation [103]. 

In female albino rats co-administered BPA and vitamin E for 3 months, vitamin E improved hepatic and kidney dysfunction by minimizing oxidative stress elicited by BPA [56,104]. In another study, while 3-week exposure of male rats to BPA increased the body weight whereas vitamin E treatment reduced the body weight than that of BPA-treated and control groups. BPA causes potentiated hepatic damage, co-administering vitamin E protected the cells from major biochemical alternations [105]. This included decrease in GSH peroxidase, GSH S-transferase, and CAT activities, and changes in glucose, cholesterol, albumin, and bilirubin levels. However, that study did not allow the evaluation of antioxidant effect of vitamin E on liver toxicity. Nonetheless, vitamin E might act synergistically with other antioxidants to protect cells exposed to BPA.

Thiobarbituric acid reactive substances (TBARS) are used to measure oxidative stress levels based on lipid peroxidation. Oxidative stress induced by BPA enhanced lipid peroxidation in albino rat, and TBARS analysis revealed increased oxidation levels affecting both enzymatic and non-enzymatic antioxidant defence systems in the rat blood [55]. After vitamin E administration, TBARS levels in the blood of BPA-induced rats were decreased, indicating reduced lipid peroxidation [55,106,107]. Vitamin E restored the enzymatic antioxidant defences by elevating the levels of SOD, CAT, and GSH in the blood of BPA-induced albino rats in vivo, perhaps by scavenging free radicals [55]. Omran et al. [108] demonstrated a significant improvement of the serum testosterone levels in BPA (325 mg/kg/d)- and vitamin E (200 mg/kg/d)-treated rats, compared with the BPA-only treated group. Vitamin E also improved histological features of the testis and prostate by manipulating Caspase3 activity [108]. 

Vitamin E may also exert a protective effect on the reproductive barrier associated with BPA exposure [109]. Indeed, vitamin E is found in Sertoli cell and pachytene spermatocyte [110], and its protective effects were also reported in other testicular cells [111]. Recently, we investigated the protective effects of vitamin E on the function of BPA-exposed spermatozoa [13]. Exposure of mouse spermatozoa to BPA (100 µM) for 6 h significantly affected sperm motility, hyperactivity, intracellular levels of key components (e.g., ATP, tyrosine phosphorylation), ROS and RNS levels, compared with the that of control cells. When BPA-exposed spermatozoa were co-incubated with vitamin E (2 mM), the above effects were alleviated, subsequently improving the ability of spermatozoa to fertilize an oocyte in an in vitro fertilization assay.

#### 4.2.3. Vitamin A

Vitamin A, the first discovered vitamin, is a fat-soluble micronutrient that plays numerous vital roles in the body. It is important for cell growth, communication, immune function, and reproduction. It can be obtained from plant and animal resources, food products, and supplements [112]. The antioxidant activity of vitamin A is diverse: it is a chain-breaking antioxidant that reacts with peroxyl radicals before they fuel peroxidation in the lipid phase and, consequently, hydroperoxide formation [113]. 

Vitamin A and its derivatives, including retinol, retinal, and retinoic acid, greatly impact cell growth and differentiation. Retinoic acid is the major biological active metabolite of vitamin A. Retinoic acid inhibits oestrogen-induced proliferation of many cell types both in vivo and in vitro [59]. Shmarakov et al. [57] reported that retinoids are stored in the liver, where they are needed for the initiation of xenobiotic elimination of BPA [57]. Further, Koda et al. [59] reported an increased uterus weight associated with abnormal morphological features of uterine cells in a rat administered BPA [59]. However, co-administration of BPA and trans-retinoic acid, another active metabolite of vitamin A, protects the uterus from abnormal weight gain by minimizing the proliferation of uterus epithelial cells. These studies convincingly demonstrate that retinoid stores in the liver are needed for the induction of xenobiotic elimination following BPA administration. 

Further, many studies showed that the human liver contains cytochromes P450 cytochrome (CYP) subfamilies involved in BPA metabolism [114,115,116]. These CYP subfamilies are reported to have retinoic acid response elements, which help to eliminate BPA from the liver. Retinoic acid acts as a tolerant that enables appropriate transcriptional and posttranscriptional responses leading to BPA biotransformation [57]. When available, retinoic acid is involved in BPA sensing and biotransformation signalling. It may disturb the oxidative damage caused by BPA [17,57]. The motility of mouse spermatozoa significantly decreases upon BPA exposure; however, the motility increases after treatment with retinoic acid in combination with BPA [58]. Therefore, vitamin A is a potential antioxidant to overcome BPA-induced stress. However, further study should be conducted to confirm the safety and efficacy for vitamin A for its clinical application.

#### 4.2.4. Melatonin

Melatonin is an endogenous hormone derived from tryptophan, which was first exposed in the vertebrate pineal gland. It regulates many important biological functions, such as sleep, circadian rhythm, reproduction, immunity, and oncostatic processes [117,118,119]. It is also involved in the maintenance of antioxidant balance, proper functioning of the immune system, and protection of the cardiovascular system. It exerts an antioxidant effect on organs and anti-apoptotic effect on cells [120]. Melatonin easily moves across the cell membrane [121], blood–brain barrier [122], and protects various biomolecules by detoxifying ROS and RNS. It also enhances the antioxidant defence system by increasing the expression of antioxidant enzymes [121,122,123,124,125].

Kobroob et al. [18] showed that 5-week BPA exposure of rat negatively impacts the kidney, affecting the renal function and increasing nitric oxide levels. It also decreases GSH and SOD activity in the kidney, leading to renal oxidative stress and potentiating lipid peroxidation. Both in vitro and in vivo, BPA affects the function of renal mitochondria, as it increases ROS levels and decreases the membrane potential [18,126]. It was reported that melatonin co-administration in BPA-treated rats protects the kidney from oxidative stress and mitochondrial damage [18]. In the kidney, melatonin restores the antioxidative enzyme activities by scavenging free radicals [127,128,129]. Based on in vitro studies, administration of melatonin before BPA administration protects the mitochondrial function in BPA-exposed rats by decreasing malondialdehyde activities and increasing GSH activities [18,127]. 

Akarca-Dizakar et al. [130] showed that melatonin treatment improves sperm motility and quality in BPA-exposed rats. They also showed a positive effect of melatonin on the adrenal and prostate function in male rats administered BPA for 2 weeks [131]. Oral administration of melatonin increased the in vitro fertilization rate, alleviating the alterations in fertility related proteins, reducing ROS levels, and preventing oocyte apoptosis induced by BPA. Melatonin also protects the uterus from deterioration induced by BPA exposure during the neonatal period in rats [132]. In another study, Wu et al. [62] reported that male rats treated with BPA did not change the weight of the reproductive organs. However, they noticed an elevated oxidative stress in testis associated with altered TBARS level and SOD activity. While BPA treated rats co-administered with melatonin, the toxic effects of BPA on testis were ameliorated [62,133].

#### 4.2.5. Quercetin (Flavonoid)

Quercetin belongs to a large group of polyphenolic compounds with a benzo-γ-pyrone structure [134,135]. It is synthesized by plants in response to microbial infection, as a hydroxylated phenolic substance [136]. As a polyphenolic flavonoid, quercetin is ubiquitous in plants and plant food sources [137]. It is one of the most abundant nutritional flavonoids found in fruits (mainly citrus fruits), green leafy vegetables, as well as many seeds, buckwheat, nuts, flowers, bark, broccoli, olive oil, apple, onion, green tea, red grape, red wine, dark cherry, and berries, such as blueberry and cranberry. It is well known for its anti-inflammatory, antihypertensive, vasodilator, anti-obesity, anti-hypercholesterolaemic, and anti-atherosclerotic activities [138]. Quercetin is a potent antioxidant because of its ability to scavenge free radicals and bind to multiple transition metal ions, thereby inhibiting excessive lipid peroxidation in the cell [138]. It reduces oxidative damage to macromolecules such as lipids and DNA [139]. Specifically, it prevents the harmful effects of oxidized low-density lipoprotein, which suggests that it may be involved in inhibiting free radical-mediated cytotoxicity and lipid peroxidation [63,139]. The antioxidant potential of quercetin is approximately four times that of vitamin E [140,141]. 

Incubation of hepatocytes with BPA results in a time-dependent cell death, along with the loss of intracellular ATP and total adenine nucleotide pools [142,143]. The antioxidative properties of quercetin may prevent ROS-associated mitochondrial dysfunction upon its co-administration with BPA, also significantly increasing the ATPase SDH activities in tissue. Similarly, quercetin potentially reduces BPA-induced oxidative stress and mitochondrial dysfunction in mouse [142,143,144].

Acid phosphate (ACP) is a marker enzyme of lysosomal integrity, and is essential for tissue repair. In mouse, oral administration of BPA results in a pronounced dose-dependent increase in ACP activity in the testis [144], suggesting cell lysis upon release of the lysosomal enzyme. BPA treatment also causes a pronounced increase in alkaline phosphatase (ALP) activity in the testis. ALP is a marker enzyme for plasma and endoplasmic reticulum. Its activity might increase because of the alteration of endoplasmic reticulum structure by BPA. By contrast, quercetin treatment significantly decreases ACP and ALP activity by preventing damage to the tissue [144].

BPA is a ligand of oestrogen receptor and enhances oestrogenic activity, which affects plasma and lipoproteins linked to the cholesterol in the blood. Quercetin has an anti-lipoperoxidative activity, ameliorating BPA-induced oxidative stress by blocking the lipid components to prevent hypercholesterolemia [63]. 

Quercetin treatment alleviates BPA-induced oxidative stress and lipid peroxidation. It also reduces lipid peroxidation in testicular tissue exposed to BPA [64]. Further, it sustains the testosterone levels, improves sperm quality, and ameliorates pathological changes associated with BPA exposure [64]. 

The chemical structure of quercetin allows ROS and RNS scavenging. The hydroxyl groups donate hydrogen and an electron to free radicals, such as hydroxyl, peroxyl, and peroxynitrite radicals, forming stable flavonoid and supporting the systems that protect the cell against cytogenic damage associated with BPA exposure. Quercetin also reverses the toxic histological and biochemical changes, and reduces the genotoxic effects of BPA. Furthermore, it reduces the rate of apoptosis associated with DNA fragmentation [64]. In addition, quercetin alleviates the reproductive toxicity induced in spermatogenic cells by other environmental oestrogenic contaminants, and enhances the expression of GSH enzymes, also protecting the cell against oxidative stress in other tissues upon BPA exposure. Finally, quercetin exerts an anti-oestrogenic effect and improves testosterone hormone levels in male rat [145]. It increases the enzymatic antioxidants SOD, CAT, and GSH peroxidase, reducing oxidative stress caused by BPA and [146].

#### 4.2.6. Lycopene (Carotenoid)

Carotenoids (also known as carotenes) are natural lipid-soluble plant pigments, such as lycopene [147]. Lycopene is an aliphatic hydrocarbon commonly found in fruits and vegetables, e.g., watermelon and tomatoes. Its antioxidant activity is stronger than that of other carotenoids and natural compounds. 

Lycopene alleviates the toxic effect of BPA in the brain [148]. BPA disrupts the cellular redox system by enhancing lipid peroxidation and lowering GSH levels in the brain cells. The brain is most sensitive to oxidative injury, because of its high lipid content, high oxidative metabolism, and low intracellular antioxidant system. BPA increases ROS levels, stimulating polyunsaturated fatty acid oxidation and hydroxyl radical consumption, which reduces GSH levels. As shown in a rat model, lycopene treatment increases GSH levels lowered by BPA in the brain tissue [148,149].

Lycopene is attacked by electrophile entities more easily than other all-natural carotenoids and is highly active against ROS. It can cross the blood–brain barrier, which explains its ability to trap ROS, protecting the cell against oxidative stress, and reducing the damage to cellular components. Lycopene protects the hippocampus against BPA-induced neurotoxicity by inhibiting the oxidative stress, improving cellular signalling, suppressing neural apoptosis, and boosting synaptic plasticity in rat [66]. While BPA deceases the levels of enzyme antioxidants, increasing oxidative stress, decrease in body/organ weight and causing testicular damage in rat, lycopene treatment of BPA-exposed rat enhances the body/organ weight, enzymatic antioxidant activity and reduces the oxidative stress. As demonstrated by histopathological analysis, lycopene also reduces testicular damage in BPA-induced rats [150].

#### 4.2.7. Synthetic Antioxidants

Synthetic antioxidants are chemically synthesized compounds that do not occur in nature. They are typically used to alleviate fat and prevent lipid oxidation. Butylated hydroxytoluene (BHT) and butylated hydroxyanisole (BHA) are the primary synthetic antioxidants with similar name, structures, and antioxidant activities, and are commonly used in fats and oils [151,152]. They have been used in human food as antioxidants since 1954 [153]. Although they are listed as safe, according to some chronic studies, high doses of BHT are tumorigenic [154]. *Tert*-butylhydroxyquinone is another synthetic antioxidant, mostly used in the feed industry. It is used as a preservative in different food products, since it does not cause food discoloration, or affect flavour or odour [155]. However, high doses are associated with some negative effects in laboratory animals, such as stomach tumours and DNA damage [156]. 

Babu et al. [17] analysed the effects of BPA and synthetic antioxidants in an in vitro experimental model. BPA elicited more pronounced oxidative stress than BHA and BHT. Further, the antioxidant activity of BHA and BHT was less than that of BPA. Accordingly, the oxidative stress caused by BPA is more pronounced than that caused by phenoxyl radicals [17]. However, more research is needed to place these synthetic antioxidants on the safe list.

#### 4.2.8. Other Natural Antioxidants

With the increased risk of deadly diseases, the use of natural substances in medication and diet as therapeutics agents is also increasing [14]. These compounds, natural antioxidants, have a strong potential to inhibit oxidative stress by scavenging free radicals, preventing lipid peroxidation, and breaking radical-mediated chain reactions. A vast majority of plants can act as possible therapeutic agents in averting human disease, including neurological diseases, diabetes, and cancer, and aging [157]. For instance, Tualang honey exerts pronounced antioxidant effects [158,159,160,161]. In humans, it protects against osteosarcoma [162], keloid fibroblast formation [163], testis damage [164], and osteoporosis [165]. BPA disrupts the oestrous cycle in rodents [166,167,168] and rodents exposed to BPA experience early-onset puberty. Zaid et al. [72] reported that treatment with Tualang honey improves the oestrus cycle and decrease the body weight in BPA-exposed rats. As another example, *Murraya koenigii* leaf extract exerts a protective effect in mouse orally administered BPA. *M. koenigii* exerts protective effects against BPA by improving sperm parameters, and decreasing lipid peroxidation and ROS levels in mouse testis [68]. Gallic acid, another natural antioxidant, is commonly found in fruits and vegetables. As reviewed by Badhandi et al. [169], it scavenges ROS and metal ions. Co-treatment of male rats with gallic acid and BPA was positively correlated with increased antioxidant enzyme activity, normalizing the gonadosomatic index used to ascertain sexual maturity with the development of testis, and reducing lipid peroxidation in the testis [69]. Ginseng is a popular traditional herb tonic in Asia [170]. Its major components are ginsenosides, useful for the treatment of ageing, immune disorders, cancer, and other conditions [136,171,172]. While in pregnant BPA-treated rats, the testosterone and progesterone levels are elevated, ginseng administration significantly decreases these levels at the end of pregnancy [70]. Further, Yang et al. [173] suggested positive chemo-preventive effects of ginseng in women expressing gynaecological complaints induced by BPA [173]. BPA substantially reduces cell viability, and compounds cytoskeleton alteration and cell apoptosis. Ginseng plays a protective role in the cell by affecting antioxidant defence mechanisms mediated by ERK1/2 MAPK signalling, thus hampering BPA stress in Sertoli cells [76]. Based on these observations, ginseng is a possible candidate for the treatment of reproductive disorders caused by environmental toxicants.

## 5. Conclusions and Future Perspectives

BPA is a focus of extensive research because of its endocrine interference and relationship with various diseases, such as diabetes, obesity, cancer, cardiovascular diseases, neurodegeneration, and reproductive abnormalities. At the molecular level, increased ROS generation, alteration of the redox balance, mitochondrial dysfunction, and modulation of cell signalling pathways are the major damaging effects induced by BPA. These changes accumulate in animals and human, and subsequently disrupt diverse physiological, metabolic, and endocrine. BPA effects can be potentiated if the exposure is linked to other health factors, such as poor diet, metabolic impairment, and coexisting disease. Approaches involving antioxidants to counter the BPA effect are being considered. Antioxidants reduce oxidative stress, lipid peroxidation, and DNA damage, and restore the overall antioxidant defence thereby prevented the harmful consequences of BPA exposure. However, further studies are required to outline their usefulness, optimal dosage, and treatment scheme for countering BPA toxicity. Besides, even many antioxidants such as GSH, vitamin C, vitamin E, N-acetylcysteine, and lipoic acid exhibit potentially beneficial effects to ameliorate BPA toxicity in vitro (reviewed in Meli et al. [19]), in many cases, their positive effect appear to be negligible in the clinical trials. Therefore, prospective controlled studies are necessary to establish a dose-dependent molecular mechanism underlying the positive impact of antioxidants to overcome BPA toxicity for the possible clinical application.

## Figures and Tables

**Table 1:** Summary of the antioxidant effects alleviating bisphenol A (BPA) toxicity: evidence from in vivo models.

BPA Dose	Target Antioxidant and Dose	Experimental Design	Mechanism of Action	Reference
5, 50, and 500 µg/kg bodyweight/d	Superoxide dismutase (SOD)	8-week-old male rats were exposed to BPA for 8 weeks	SOD levels in the liver were decreased by the higher dose tested	[44]
0, 2, 10, and 50 mg/kg bodyweight/d		50-d-old male rats were treated with BPA for 30 d	SOD levels were reduced by the highest concentration tested	[45]
20 and 100 mg/kg bodyweight/d		Male albino rats were exposed to BPA for 30 d	SOD levels decreased in the liver and testis	[46]
5, 50, and 500 µg/kg bodyweight/d	Catalase (CAT)	8-week-old male rats were exposed to BPA for 8 weeks	CAT levels in the liver were reduced upon exposure to the highest dose	[44]
0, 2, 10, and 50 mg/kg bodyweight/d		50-d-old male rats were treated with BPA for 30 d	CAT levels in the liver were reduced in a dose-dependent manner	[45]
50 and 25 mg/kg bodyweight/d		Male mice were given BPA intraperitoneally for 5 d	CAT activity was significantly reduced in the liver	[47]
20 and 100 mg/kg bodyweight/d	Glutathione (GSH)	Male rats were exposed to BPA for 30 d	GSH levels decreased in the testis and liver	[46]
0.1, 1, 10, and 50 mg/kg bodyweight/d		Male rats were given BPA for 4 weeks	GSH levels decreased in the liver and reactive oxygen species (ROS) levels increased	[48]
50 and 25 mg/kg bodyweight/day		Male mice were given BPA intraperitoneally for 5 d	GSH levels decreased in the kidney but were unchanged in the liver	[47]
5, 50, and 500 µg/kg bodyweight/d	Uric acid	6-week-old male mice were given BPA for 8 weeks	BPA decreased hepatic uric acid levels	[49]
25 and 10 mg/kg bodyweight/d		Adult male rats were administered BPA for 6 and 10 weeks	Both dosages increased uric acid levels in the kidney leading to its malfunction	[50]
0.2, 2, and 20 µg/kg bodyweight/d	Vitamin C, 40 mg/kg bodyweight/d	Male rats were exposed to BPA for 60 d	Vitamin C had a protective effect on the epididymis in BPA-exposed rats	[51]
60 µg/kg bodyweight/day	Vitamin C, 150 mg/kg bodyweight/d	Female rats were co-administered BPA and Vitamin C for 20 d	BPA reduced the volume of the ovary cortex and medulla, and the volume of oocyte; vitamin C treatment alleviated these effects	[52]
25 mg/kg bodyweight/d	Vitamin C, 60 and 5.5 mg/kg bodyweight/d	Rats co-administered BPA and Vitamin C for 6 weeks	Vitamin C co-treatment reduced BPA hepatotoxicity and nephrotoxicity	[53]
5, 50, 100 μg/100 g bodyweight /d	Vitamin E, 4 mg /100 g bodyweight/d	3-month-old male rats were co-administered BPA and Vitamin E for 3 months	Vitamin E protected the testicular cells and epididymal sperm from apoptosis in BPA-exposed rats	[54]
20 mg/kg bodyweight/d	Vitamin E, 200 mg/kg bodyweight/d	Male rats were treated for 15 d	BPA enhanced lipid peroxidation in the blood; this was alleviated by vitamin E	[55]
20 mg/kg bodyweight/d	Vitamin E, 0.57 mg /100 g bodyweight/d	6 to 8-week-old female rats were treated for 15 d	Vitamin E restored the function of hypothalamus–pituitary–gonadal axis in BPA-exposed rats	[56]
50 mg/kg bodyweight/d	Vitamin A, 3000 IU	10 to 12-week-old male mice were treated for 3 d	Vitamin A co-administration alleviated BPA toxicity in the liver	[57]
50 µg/d	Vitamin A, 100 IU	Male mice were co-administered BPA and Vitamin A for 5 d from the day of birth	Treatment with vitamin A increased sperm motility	[58]
100 mg/kg bodyweight/d	Vitamin A, 5 mg/kg bodyweight/d	10 to 11-week-old female rats were co-administered BPA and Vitamin A for 3 d	Vitamin A minimized epithelium cell proliferation in BPA-exposed mice	[59]
10 mg/kg bodyweight/d	Melatonin, 10 mg/kg bodyweight/d	Male mice were co-administered BPA and melatonin for 14 d	Melatonin reduced mitochondrial toxicity in the testis of BPA-exposed rats	[60]
50 mg/kg bodyweight /d	Melatonin, 10 mg/kg bodyweight/d	8-week-old male rats were co-administered BPA and melatonin for 6 weeks	Melatonin improved GSH, SOD, CAT, malondialdehyde, and H_2_O_2_ levels in mice co-treated with BPA	[61]
200 mg/kg bodyweight/d	Melatonin, 10 mg/kg bodyweight/d	8-week-old male rats were co-administered for 10 d	Melatonin repaired DNA damage in male germ cells by suppressing the oxidative stress in BPA-treated rats	[62]
50 mg/kg bodyweight/d	Quercetin, 50 mg/kg bodyweight/d	Adult male rats were co-administered BPA and quercetin for 52 d	Quercetin reduced plasma total cholesterol, triglyceride, and low-density lipoprotein cholesterol levels in BPA-treated rats	[63]
50 and 100 mg/kg bodyweight/d	Quercetin, 10 mg/kg bodyweight/d	Adult male mice were co-administered BPA and quercetin for 6 weeks	Quercetin reduced abnormal testis weight, and improved sperm quality and quantity in BPA-exposed mice	[64]
10 mg/kg bodyweight /day	Lycopene, 10 mg/kg bodyweight/d	Male rats were co-administered BPA and lycopene for 3 months	Lycopene enhanced LYC glucose haemostasis, fat mass, and thyroid hormone levels, and decreased oxidative stress in BPA-treated rats	[65]
50 mg/kg bodyweight/d	Lycopene, 10 mg/kg bodyweight/d	8-week-old male rats were gavaged 3 d a week for 6 weeks	BPA induced neurotoxicity in the hippocampus by eliciting oxidative stress; lycopene inhibited this effect	[66]
500 mg/kg bodyweight/d	Lycopene, 20 mg/kg bodyweight/d	8-week-old female and male mice were exposed to BPA from PD8 to PD14, and to lycopene from PD1 to PD7	Lycopene reduced the negative effect of BPA on pregnant mice	[67]
1 mg/kg bodyweight/d	*Murraya koenigii*, 200 mg/kg bodyweight/d	Male mice were treated for 8 weeks	*M. koenigii* extract recovered sperm parameters and reduced ROS, lipid peroxidation, and apoptotic protein levels in BPA-induced mice	[68]
10 mg/kg bodyweight/d	Gallic acid, 20 mg/kg bodyweight/d	Male albino rats were exposed to BPA and gallic acid for 45 d	Gallic acid reduced the chronic stress caused by BPA, by increasing antioxidant enzyme levels and lowering lipid peroxidation	[69]
150 mg/kg bodyweight/d	Ginseng, 200 mg/kg bodyweight/d	Adult female albino rats were given BPA and ginseng from pregnancy day 0 until day 20	Ginseng reduced testosterone and progesterone levels in BPA-treated pregnant rats	[70]
10 mg/kg bodyweight/d	Tualang honey, 200 mg/kg bodyweight/d	Female rats were treated for 6 consecutive weeks	BPA-induced uterine disturbance was lessened by Tualang honey owing to its phytochemical properties	[71]
		21-year-old pre-pubertal female rats were treated for 6 consecutive weeks	Tualang honey decreased ovarian toxicity by reducing morphological abnormalities and enhancing the normal oestrous cycle	[72]

**Table 2:** Summary of antioxidant effects alleviating bisphenol A (BPA) toxicity: evidence from in vitro models.

BPA Dose	Target Antioxidant and Dose	Experimental Design	Mechanism of Action	Reference
0, 1,10, and 100 µg/L	Superoxide dismutase (SOD)	Rat sperm and testicular tissues were incubated with BPA for 2 h	BPA exposure increased SOD activity by eliciting oxidative stress	[73]
0.1 to 500 µg/mL	SOD	Erythrocytes were treated with BPA for 1, 4, or 24 h	SOD activity was reduced in BPA-incubated erythrocytes	[74]
	Catalase		Catalase levels were reduced by increased number of hydrogen peroxide ions generation by BPA	
	Glutathione (GSH)		BPA-treated cells showed decrease GSH levels	
100 µM	GSH, 5 mM	Mouse spermatozoa were treated with BPA and glutathione for 6 h	GSH reduced oxidation and compromised acrosome integrity in BPA-exposed spermatozoa	[13]
100 µM	Vitamin C, 100 µM	Mouse spermatozoa were treated with BPA and glutathione for 6 h	Motility of the spermatozoa was increased and stress reduced upon vitamin C treatment	[13]
100 µM	Vitamin E, 2 mM	Mouse spermatozoa were treated with BPA and glutathione for 6 h	Vitamin E restored fertilization and embryo development capacity of BPA-treated cells	[13]
125 µM	Melatonin, 0.5 µM	Kidney mitochondria were pre-incubated with melatonin for 5 min and then exposed to BPA for 15 min	Melatonin protected mitochondrial function, reduced malondialdehyde levels, and increased GSH levels in BPA-exposed cells	[18]
25–150 µg/mL	Quercetin, 10–50 µg/mL	Blood samples	BPA reduced the activity of enzymatic antioxidants while quercetin ameliorated this effect	[75]
50 µM	Synthetic antioxidants butylated hydroxytoluene (BHT) and butylated hydroxyanisole (BHA) with rifampicin, 100 µM	100 µM rifampicin was mixed with 0.2 µM horseradish peroxidase (HRP), 100 µM H_2_O_2_, and 50 µM phenolic compounds BHA, BHT, or BPA in 1.0 ml of 0.1 M phosphate buffer, pH 5.5; oxidation of rifampicin was then monitored for 5 min	BPA had the highest pro-oxidant activity in BHA and BHT in a rifampicin solution, which had a protective effect against oxidative stress	[17]
10 µM	Synthetic antioxidants BHT and BHA with NADPH, 100 µM, in 1.0 mL of phosphate buffer	100 µM reduced NADPH was incubated with 0.2 µM HRP, 10 µM H_2_O_2_, and 10 µM BHA, BHT, or BPA in 1.0 ml of 10 mM phosphate buffer, pH 7.0, containing 50 µM DTPA; oxidation of NADPH was then monitored continuously for 5 min	BPA showed higher oxidation of NADPH compared to synthetic antioxidants	
10, 20 µM	Ginseng, 75 µg/mL	Sertoli cells were cultured for 6, 24, or 48 h	Ginseng increased the activity of antioxidative enzymes, and reduced cell apoptosis and lipid peroxidation in BPA-treated cells	[76]
100 µM	Ginseng, 10, 25, and 50 µg/mL	Leydig and Sertoli cells were pre-treated with ginseng for 1 h and then with BPA for 24 h	Ginseng prevented apoptotic cell death and its anti-apoptotic ability could be useful for cellular defence	[77]
